# Revealing Structural Brain-Cognition Relationships in Children: A Comparison of Morphometric Similarity and INverse Divergence Networks

**DOI:** 10.1007/s12021-025-09764-z

**Published:** 2026-01-08

**Authors:** Shuning Han, Hao Jia, Gemma Vilaseca, Núria Vilaró, Feng Duan, Zhe Sun, Cesar F. Caiafa, Jordi Solé-Casals

**Affiliations:** 1 Data and Signal Processing Research Group, University of Vic-Central University of Catalonia, Vic, Catalonia 08500 Spain; 2 School of Medicine, Nankai University, Tianjin, 300355 China; 3 Tianjin Key Laboratory of Brain Science and Intelligent Rehabilitation, Nankai University, Tianjin, 300355 China; 4 Psychological Department, Oms and Prat school, Fundació Catalunya - La Pedrera, Manresa, Catalonia 08243 Spain; 5 Oms Foundation, Manresa, Catalonia 08243 Spain; 6 Faculty of Health Data Science, Juntendo University, Urayasu, Chiba 279-0013 Japan; 7 Instituto Argentino de Radioastronomía-CCT La Plata, CONICET / CIC-PBA / UNLP, V. Elisa, 1894 Argentina; 8 Department of Psychiatry, University of Cambridge, Cambridge, CB20SZ UK

**Keywords:** Structural magnetic resonance imaging, Gifted children, Morphometric similarity network, Morphometric INverse divergence, Hemispheric connectivity, Topological features

## Abstract

The study of structural brain networks (SBNs) offers critical insights into brain-cognition relationships. However, a comprehensive comparison of these methods in terms of their topological properties, cognitive relevance, and sensitivity to connection density remains lacking. This study compares two types of individual-level SBNs–morphometric similarity networks (MSNs) and morphometric inverse divergence (MIND) networks–by analyzing their associations with cognitive performance using sMRI data from 29 male children. Group- and individual-level analyses were conducted to evaluate differences in hemispheric connectivity, topological features, and their correlations with cognitive performance across different connection densities. In our analyses, a connection density of $p=0.05\sim 0.15$ appeared optimal for stabilizing network properties and maximizing cognitive correlations in both MSN and MIND. Moreover, advanced network segregation and integration metrics (such as local efficiency and node versatility, along with their global summaries) demonstrated greater sensitivity to cognitive performance. However, MSNs appeared to provide a more reliable framework, demonstrating more stable associations across connection densities in topological and hemispheric dimensions. Specifically, higher cognitive performance may be linked to stronger left intra-hemispheric connectivity, weaker inter-hemispheric connectivity, and more modular network organization–consistent with established theories of hemispheric specialization and efficient modularity. In contrast, MIND networks exhibit reduced effectiveness and stability across metrics and densities in our data. These preliminary insights enhance our understanding of brain-cognition relationships and provide practical guidelines for parameter selection and metric identification in network-based cognitive analyses.

## Introduction

Cognitive neuroscience seeks to understand the mechanisms underlying human cognition. A promising approach involves studying brain networks, which map connectivity between distinct regions to unravel the neural basis of cognition and behavior. Advances in neuroimaging, such as structural magnetic resonance imaging (sMRI) and diffusion MRI, have enabled the construction of structural brain networks (SBNs), providing insights into the anatomical connectivity of the brain in vivo (Sebenius et al., 2025; Genon et al., 2022; Lo et al., 2011). Graph theoretical analysis further characterizes the topological properties of SBNs, linking them to biological and cognitive attributes, such as gender (Sun et al., 2015) and cognitive performance (Park et al., 2013).

Two primary methods for constructing SBNs exist: tractography from diffusion-weighted imaging (DWI) and structural covariance networks (SCNs) derived from sMRI. This study focuses on SCNs, using sMRI to construct brain networks. Traditional SCNs typically compute group-level covariances of a single morphometric feature, such as cortical thickness (CT) (Solé-Casals et al., 2019). However, this approach provides limited insights into individual variability in brain network organization. To address this limitation, recent innovations have introduced individual-level SCNs, incorporating multiple morphometric features into analyses (Kong et al., 2015; Li et al., 2017; Yu et al., 2018; Seidlitz et al., 2018; Li et al., 2021; Sebenius et al., 2023; Sun et al., 2024a, b; Vuksanović, 2022). These individual-level SCNs, such as morphometric similarity networks (MSNs) (Seidlitz et al., 2018) and morphometric inverse divergence (MIND) networks (Sebenius et al., 2023), allow for a more detailed and personalized understanding of brain structure and its relationship with cognitive and behavioral factors. The MSN (Seidlitz et al., 2018) transforms each individual’s set of multimodal MRI features into a morphometric similarity matrix of pairwise inter-regional correlations of morphometric feature vectors. In contrast, the MIND (Sebenius et al., 2023) estimates the similarity between cortical areas at the individual level by the symmetric Kullback-Leibler (KL) divergence (Jeffreys, 1973) between their multivariate distributions of vertex morphometric features. Although various methods have shown substantial promise in revealing morphometrically relevant patterns in brain networks, very few published studies have systematically compared brain networks with cognitive performance in terms of their topological properties and cognitive relevance. Such a comprehensive comparison remains crucial for clarifying their relative advantages, limitations, and applicability across different research contexts. Moreover, understanding how these methods converge or diverge in representing SBNs can offer critical insights into optimizing network construction for cognitive neuroscience.

Another challenge in SBN studies lies in thresholding weighted networks to enhance interpretability. Weighted networks, which encode the strength of connections between brain regions, are often converted into sparse networks by thresholding edge weights to enhance interpretability and simplify graph analysis. However, this thresholding process can significantly alter network topology, raising concerns about the consistency and reproducibility of results. Furthermore, no universally accepted thresholding criteria currently exist, making it difficult to standardize methods across studies (Chung, 2019; Liu et al., 2017). For example, a connection density of 0.01 was adopted in Sebenius et al. (2023), while a connection density of 0.1 was used in Seidlitz et al. (2018); Solé-Casals et al. (2019). Such disparities in connection density can lead to inconsistent network topologies, making it challenging to compare results and derive generalizable conclusions. Therefore, systematically evaluating the impact of connection density is crucial, especially in comparative studies of different network construction methods.

By addressing these challenges, this research seeks to comprehensively compare MSN and MIND networks in a cohort of 29 male children, focusing on their topological properties and cognitive associations across different connection densities. We analyze both group- and individual-level data, mainly focusing on:

- The impact of connection density on network stability and cognitive associations,
- The role of hemispheric connectivity patterns in cognitive performance, and
- The relationships between topological features and cognitive performance.

The structure of this paper is as follows: Section “Materials and Methods” describes the dataset and methodologies used. Section “Results” details the analysis results, followed by a discussion in Section “Discussion”. Finally, Section “Conclusion” provides the conclusions of the study.

## Materials and Methods

This section outlines the data acquisition, network construction, and analytical methodologies employed in this study. Cognitive test data and sMRI data were obtained from 29 male children, who were divided into a control group (CG) and a gifted group (GG). Two individual-level brain networks–MSN and MIND–were constructed for subsequent comparative analysis. Group-level comparisons were performed between CG and GG average networks, focusing on hemispheric connectivity and nodal topological features across different connection densities. At the individual level, Spearman correlation analysis was conducted to assess the relationships between global network metrics (including seven topological and five hemispheric metrics) and cognitive performance scores.

### Data

#### Participants

In this study, we use the dataset consisting of sMRI and cognitive tests from 29 healthy right-handed male children with no history of psychiatric or neurological disorders. This dataset was previously used for group-level SCN analysis in our earlier work (Solé-Casals et al., 2019). The raw (anonymized) MRI data are available in the OpenNeuro repository at https://openneuro.org/datasets/ds001988.

Participants were divided into two groups: the control group (CG, 14 subjects) and the gifted group (GG, 15 subjects). Participant details are presented in Table 1. The table indicates no notable age differences between groups but reveals significant differences in full-scale IQ scores.

**Table 1:** Participant information

Groups	CG (Mean ± SD)	GG (Mean ± SD)
Age	$12.53 \pm 0.77$	$12.03\pm 0.54$
Full-scale IQ	$122.71 \pm 3.89$	$148.80 \pm 2.93$

#### sMRI Data

Participants underwent MRI scanning using a 3T scanner. High-resolution T1-weighted images were acquired with the MPRAGE 3D protocol (TR = 2300*ms*; TE = 3*ms*; TI = 900*ms*; FOV = $244\times 244 mm^{2}$; 1*mm* isotropic voxel). In this study, we adopted the sMRI preprocessing method outlined in prior research (Solé-Casals et al., 2019). FreeSurfer v5.3 was used for preprocessing to estimate cortical thickness (CT) from a three-dimensional cortical surface model based on intensity and continuity information (Fischl et al., 2000). Cortical reconstructions were independently reviewed by two experienced researchers to ensure adherence to quality control criteria. Each brain was parcellated into 308 regions ($R=308$; approximately $500\ \textrm{mm}^2$ each) using the standard FreeSurfer fsaverage template and a backtracking algorithm (Romero-Garcia et al., 2012; Solé-Casals et al., 2019), which further subdivides the regions defined in the Desikan-Killiany atlas (Desikan et al., 2006). Among them, 152 regions belong to the left hemisphere ($N_L=152$) and 156 to the right hemisphere ($N_R=156$). The surface-based (non-linear) registration by the FreeSurfer command mri_surf2surf was then applied to warp the parcellation from the standard template to each individual’s native MPRAGE space. This method is recommended for superior cortical landmark alignment and for avoiding the introduction of age-associated bias (Ghosh et al., 2010).

In this work, we chose five morphometric features for both MIND network and MSN construction to maintain consistency for comparison: surface area (SA), gray matter volume (GMV), cortical thickness (CT), mean curvature (MC), Gaussian curvature (GC). Specifically, while regional-level morphometric features were employed for MSN estimation, vertex-level morphometric features of each region were utilized for MIND estimation in each subject.

### Methods

In this study, we construct and analyze two types of individual-level brain networks–the morphometric similarity network (MSN) and the morphometric inverse divergence (MIND) network–for comparative analysis at both group and individual levels. In the group analyses, we compare the average brain networks of CG and GG groups across MSN and MIND networks, including intra-/inter-hemispheric distinctions, effects of varying connection densities, and nodal topological features. In the individual cognitive analysis, we assess the correlation between the global topological features and the scores of cognitive indices using Spearman correlation methods.

**Fig. 1:**
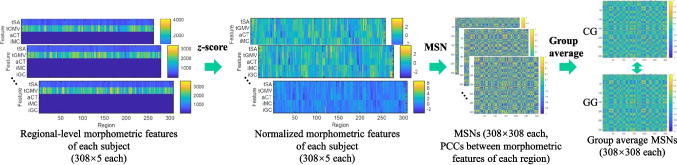
MSN construction and processing for group average MSNs. Five morphometric features (tSA, tGMV, aCT, iMC, iGC) are extracted from each brain region, resulting in $308\times 5$ features per sample. These features are normalized using the *z*-score method. MSNs ($308\times 308$) are then computed by calculating the PCCs between the normalized features of all region pairs. Finally, group average MSNs are generated for both CG and GG

#### Network Construction

In this study, we construct two types of individual-level brain networks for comparative analysis: the MSN (Li et al., 2017; Seidlitz et al., 2018) and the MIND network (Sebenius et al., 2023). Both network types are analyzed as weighted networks, which more accurately capture brain features compared to binary networks (Qi et al., 2015). To ensure network connectivity while controlling for sparsity, we employ a minimum spanning tree (MST)-based thresholding approach (Van Wijk et al., 2010) across a range of connection densities *p*. This method guarantees that all thresholded networks remain fully connected while preserving the strongest connections.

- **Morphometric Similarity Network (MSN).**

The MSN (Seidlitz et al., 2018) transformed each individual’s set of multimodal MRI features into a morphometric similarity matrix of pairwise inter-regional correlations of morphometric feature vectors. In this study, as depicted in Fig. 1, a set of $\eta$ ($\eta =5$) morphometric features ($308\times 5$ for each sample) derived from any T1-weighted MRI scans: total SA (tSA), total GMV (tGMV), average CT (aCT), integrated rectified MC (iMC), integrated rectified GC (iGC), was employed to construct MSNs. It has been demonstrated that the MSNs based on these five features are similar to MSNs utilizing a broader array of features (Seidlitz et al., 2018), with tSA, tGMV, aCT, and iGC identified as the most discriminative features (Zhang et al., 2021). In accordance with prior studies (Li et al., 2017; Seidlitz et al., 2018), each feature vector is standardized by the *z*-score values prior to the correlation calculation. The morphometric similarity between each possible pair of regions was estimated by the Pearson’s correlation coefficient (PCC) between their normalized morphometric feature vectors, resulting in a $308\times 308$ MSN for each sample. As commonly acknowledged, a PCC value close to -1 denotes anti-correlation between the pair of features, while a PCC value close to 1 denotes strong correlation between the pair of features (Heinsfeld et al., 2018). Hence, the diagonal elements of MSN equal to 1. However, we uniformly assign NaN values to the diagonal elements of MSNs.

- **Morphometric INverse Divergence (MIND) network.**

**Fig. 2:**
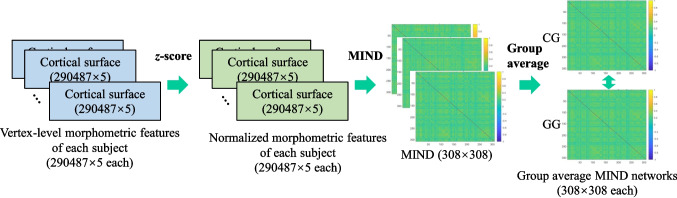
MIND network construction and processing for group average MIND networks. Five features (SA, GMV, CT, MC, GC) of each vertex normalized by *z*-score are used to compute the similarity between cortical regions at the individual level based on the symmetric KL divergence, through this, we can get the MIND networks ($308\times 308$) for each sample. Finally, the group average MIND networks are generated for both groups CG and GG

The MIND (Sebenius et al., 2023) estimates the similarity between cortical areas at the individual level by the symmetric KL divergence (Jeffreys, 1973) between their multivariate distributions of MRI vertex features. In this study, the MIND network’s input comprises mesh representations of cortical surfaces derived from T1-weighted MRI scans. Each surface is delineated by a set of 290,487 vertices. To maintain consistency with MSN methodology for comparison, each vertex is characterized by five structural MRI features: SA, GMV, CT, MC and GC. In Sebenius et al. (2023), as depicted in Fig. 2, *z*-score was employed to standardize each feature across all vertices in the brain prior to parcellating the data into vertex-level distributions. Thus, each surface can be described by a set of vertices $\boldsymbol{v}_i \in \mathcal {V}$, where $\boldsymbol{v}_i$ represents the vector of $\eta$ ($\eta =5$) structural features of the *i*th vertices in the surface. $\boldsymbol{v}_i$ in a surface can be grouped into $R (R=308)$ regions according to the parcellation, such that $\mathcal {V}=\{\{\mathcal {V}_1\},\ldots ,\{\mathcal {V}_R\}\}$. Given regions *a* and *b*, vertices $\mathcal {V}_a$ and $\mathcal {V}_b$, with true multivariate distributions $P_a$ and $P_b$, the KL divergence between $P_a$ and $P_b$ is defined as:

1 $$\begin{aligned} d_{KL}(P_a\parallel P_b)=\int _{\mathbb {R}^{\eta }}P_a(\boldsymbol{x})\log {\frac{P_a(\boldsymbol{x})}{P_b(\boldsymbol{x})}} d\boldsymbol{x} \ge 0 \end{aligned}$$

The *k*-nearest ($k=1$) neighbor divergence approximation (Pérez-Cruz, 2008) was used to estimate $d_{KL}(P_a\parallel P_b)$:

2 $$\begin{aligned} \hat{d}_{KL}(P_a\parallel P_b)=-\frac{\eta }{n}\sum \limits _{i=1}^{n}\log {\frac{r_k(\boldsymbol{x}_i)}{s_k(\boldsymbol{x}_i)}}+\log {\frac{\parallel \mathcal {V}_b\parallel }{\parallel \mathcal {V}_a \parallel -1}} \end{aligned}$$

Here, $r_k(\boldsymbol{x}_i)$ and $s_k(\boldsymbol{x}_i)$ represent the Euclidean distances of $\boldsymbol{x}_i$ to the *k*-th most similar vertex of $\boldsymbol{x}_i$ in $\mathcal {V}_a$ (with the sample $\boldsymbol{x}_i$ removed) and $\mathcal {V}_b$, respectively. A symmetric measure of KL divergence is then defined as:

3 $$\begin{aligned} \begin{aligned} \hat{d}(P_a,P_b)=\max (\hat{d}_{KL}(P_a\parallel P_b),0) +\max {\hat{d}_{KL}(P_b\parallel P_a),0)} \end{aligned} \end{aligned}$$

KL divergence is always greater than or equal to zero, and it equals zero only when the two distributions are identical. Finally, the KL divergence for regions a and b was transformed by Eq. 4 to estimate the inter-areal MIND similarity. The values of MIND are bounded between 0 and 1, with higher values denoting a stronger degree of similarity.

4 $$\begin{aligned} MIND(a,b)=\frac{1}{1+\hat{d}(P_a,P_b)} \end{aligned}$$

- **Brain network sparsification.**

Based on the fundamental premise that the brain operates as an integrated system where no neural elements should be completely isolated (Fornito et al., 2016), we employ a **minimum spanning tree (MST)** based thresholding method (Van Wijk et al., 2010; Tewarie et al., 2015) to sparsify brain networks at varying connection densities *p* while ensuring the graphs remain node connected. This approach, widely used in brain network analysis (Alexander-Bloch et al., 2010; Solé-Casals et al., 2019; Han et al., 2024), utilizes Kruskal’s algorithm to construct an MST that connects all *R* nodes with $R-1$ edges, effectively maximizing the sum of edge weights in weighted networks. The thresholding procedure starts with the MST as a backbone, to which additional edges are added to achieve the desired density *p*, defined as the ratio of actual edges to the maximum possible edges $(R-1)R/2$. To ensure methodological consistency across different network types, when computing MST-based graphs for MSNs, negative values are set to zero, aligning with MIND networks where all values are inherently non-negative. Here, we denote $\mathcal {M}_p$ as the MST-based thresholding network with connection density *p* derived from brain network $\mathcal {A}$.

For a symmetric brain network $\mathcal {A}$, the MST-based thresholding procedure is implemented as follows:

1. MST construction:
  Compute distance matrix: $\mathcal {D}_{ij} = \frac{1}{\mathcal {A}_{ij}}$ (for $i \ne j$), $\mathcal {D}_{ii} = 0$
  Build MST using Kruskal’s algorithm: $E_{\text {MST}} = \text {Kruskal\_MST}(\mathcal {D})$
2. Edge selection:
  Sort edges in descending order: $E_{\text {sorted}} = \text {sort}(\{\mathcal {A}_{ij} \mid i < j\}, \text {descending})$
  Calculate target number of edges: $k_{\text {target}} = \left\lfloor p \cdot \frac{R(R-1)}{2} \right\rfloor$
  Determine additional edges to add: $k_{\text {add}} = k_{\text {target}} - |E_{\text {MST}}|$
  Construct selected edge set: $E_{\text {selected}} = E_{\text {MST}} \cup \{\text {top } k_{\text {add}} \text { edges from } E_{\text {sorted}} \setminus E_{\text {MST}}\}$
3. Output matrix construction:
  Define selection matrix:
    $$\mathcal {I}_S(i,j) = {\left\{ \begin{array}{ll} 1 & \text {if } (i,j) \in E_{\text {selected}} \\ 0 & \text {otherwise} \end{array}\right. }$$
  Compute final adjacency matrix: $\mathcal {M}_p = \mathcal {A} \circ \mathcal {I}_S$, where $\circ$ denotes the Hadamard product.

#### Group Analyses for MSNs and MIND Networks

We conducted comprehensive group-level comparisons between the CG and GG groups using average brain networks derived from both MSN and MIND frameworks (Figs. 1, 2). Our analysis encompassed two key dimensions: hemispheric connectivity patterns and nodal topological features, both evaluated across different connection densities.

Particularly, we use the **(mean) signed Euclidean distance (SED)** to evaluate the difference between the two groups across each vector metric. The SED directly quantifies differences at corresponding nodes/edges, with positive values indicating higher means in GG compared to CG, and negative values indicating the reverse.

- **Hemispheric connectivity analysis.**

To investigate hemispheric connectivity differences, we first rearrange the brain networks accordingly. For an MST-based network $\mathcal {M}_p$, we extract three hemispheric connection types: left intra-hemispheric (LL), right intra-hemispheric (RR), and inter-hemispheric (LR). These are formally defined as:

5 $$\begin{aligned} \begin{aligned} \boldsymbol{m}_{LL}^p = \left\{ \mathcal {M}^{ij}_p |i< j; i,j \in N_L \right\} , \\ \boldsymbol{m}_{RR}^p = \left\{ \mathcal {M}^{ij}_p | i < j; i,j \in N_R \right\} , \\ \boldsymbol{m}_{LR}^p = \left\{ \mathcal {M}^{ij}_p | i \in N_L; j \in N_R \right\} . \end{aligned} \end{aligned}$$

where:

- $N_{L}$ and $N_{R}$ are the node sets in left and right hemisphere, respectively.
- $\mathcal {M}^{ij}_p$ is the edge between node *i* and node *j* in the network $\mathcal {M}_p$.
- $\boldsymbol{m}_{LL}^p$, $\boldsymbol{m}_{RR}^p$, and $\boldsymbol{m}_{LR}^p$ denote the vectors containing the connections for left intra-hemispheric, right intra-hemispheric, and inter-hemispheric edges, respectively.

(1) *Effects of varying connection densities.*

Given that all nodes in an MST-based network $\mathcal {M}_p$ are connected, the minimum connection density *p* of MST-based network $R=308$ nodes is $(R-1)/[R(R-1)/2] = 0.0065$. In this study, we examine 16 connection densities as follows: $p \in \{0.0065, 0.01, 0.05, 0.1, 0.15, 0.2, 0.25, 0.3, 0.35, 0.4, 0.5, 0.6, 0.7, 0.8, 0.9, 1\}$.

To address scale disparities between MSN and MIND networks, we first normalize their average MST-based networks using Min-Max scaling across both groups to preserve relative magnitudes:

6 $$\begin{aligned} \left[ \hat{\mathcal {M}}^C_p, \hat{\mathcal {M}}^G_p\right] = \text {MinMax}_{[0,1]}\left( \left[ \mathcal {M}^C_p, \mathcal {M}^G_p\right] \right) . \end{aligned}$$

where:

- $\hat{\mathcal {M}}^C_p$ and $\hat{\mathcal {M}}^G_p$ represent the normalized networks for the CG and GG average at connection density *p*, respectively.
- MinMax scaling normalizes the values in the range [0, 1].

For each hemispheric connection type (LL, RR, LR) and density *p*, we calculate the mean SED ($\bar{D}_p$) between the normalized CG and GG average networks, separately for MSN and MIND networks:

7 $$\begin{aligned} \bar{D}_{LL}^p = {\text {sgn}}\left( \mu (\hat{\boldsymbol{m}}_{LL}^{G,p}) - \mu (\hat{\boldsymbol{m}}_{LL}^{C,p}) \right) \cdot \frac{\left\| \hat{\boldsymbol{m}}_{LL}^{G,p} - \hat{\boldsymbol{m}}_{LL}^{C,p} \right\| _2}{|\hat{\boldsymbol{m}}_{LL}^{G,p}|} \end{aligned}$$

where:

- $\hat{\boldsymbol{m}}_{LL}^{G,p}$ and $\hat{\boldsymbol{m}}_{LL}^{C,p}$ are the left intra-hemispheric connections from the normalized GG ($\hat{\mathcal {M}}^G_p$) and CG ($\hat{\mathcal {M}}^C_p$) MST-based networks with connection density *p* following the Eq. 5.
- $\mu (\cdot )$ denotes the mean value.
- $\left\| \cdot \right\| _2$ is the Euclidean norm.
- $|\cdot |$ represents the length of the vector.
- ${\text {sgn}}(\cdot )$ is the sign function preserving directionality of group differences.

Analogous calculations yield $\bar{D}_{RR}^p$ (right intra-hemispheric) and $\bar{D}_{LR}^p$ (inter-hemispheric) mean SEDs for both MSN and MIND.

(2) *Hemispheric connection strength comparison.*

For a given MST-based network $\mathcal {M}_p$, we compute the strengths for each hemispheric connection type (LL, RR, LR):

8 $$\begin{aligned} {\begin{matrix} S_{LL}^p = \frac{\sum {\boldsymbol{m}_{LL}^p}}{|\boldsymbol{m}_{LL}^p|}, \\ S_{RR}^p = \frac{\sum {\boldsymbol{m}_{RR}^p}}{|\boldsymbol{m}_{RR}^p|}, \\ S_{LR}^p = \frac{\sum {\boldsymbol{m}_{LR}^p}}{|\boldsymbol{m}_{LR}^p|}. \end{matrix}} \end{aligned}$$

where $|\boldsymbol{m}_{LL}^p|$, $|\boldsymbol{m}_{RR}^p|$ and $|\boldsymbol{m}_{LR}^p|$ represent the number of connections in the corresponding connection types.

For group-level comparison, we compute these strength measures of CG and GG at connection density $p=0.1$ for both MSN and MIND.

- **Analysis of nodal topological features.**

Eight nodal topological features (including node degree $\boldsymbol{F}_{nd}$, node strength $\boldsymbol{F}_{ns}$, eigenvector centrality $\boldsymbol{F}_{ec}$, participation coefficient $\boldsymbol{F}_{pc}$, node (betweenness) centrality $\boldsymbol{F}_{nc}$, local efficiency $\boldsymbol{F}_{le}$, clustering coefficient $\boldsymbol{F}_{cc}$, node versatility $\boldsymbol{F}_{nv}$), are adopted to characterize the nodal topological organization of brain networks. Each nodal topological feature is a vector with a length equivalent to the number of regions ($R=308$ in this study).

1. The node degree $\boldsymbol{F}_{nd}(i)$ refers to the number of connections (or edges) that node *i* has to other nodes in a graph.
2. The node strength $\boldsymbol{F}_{ns}(i)$ is the sum of weights of links connected to node *i*.
3. The eigenvector centrality (Newman et al., 2010) of node *i*, $\boldsymbol{F}_{ec}(i)$, is the *i*-th element in the eigenvector corresponding to the largest eigenvalue of the adjacency matrix.
4. The participation coefficient $\boldsymbol{F}_{pc}(i)$ (Guimera & Nunes Amaral, 2005) measures how the connections of node *i* are distributed across various modules by representing the proportion of its connectivity allocated to each module: $\boldsymbol{F}_{pc}(i)= 1 - \sum _{s=1}^{N_M} (\kappa _{is} / \kappa _i)^2$, where $N_M$ is the number of modules in the network, $\kappa _{is}$ is the total weight of connections between node *i* and all nodes in module *s*, and $\kappa _i$ is the strength of node *i*.
5. The node (betweenness) centrality $\boldsymbol{F}_{nc}(i)$ (Brandes, 2001) quantifies the proportion of shortest paths between all node pairs in the network that pass through a given index node *i*.
6. The local efficiency $\boldsymbol{F}_{le}(i)$ (Rubinov & Sporns, 2010) quantifies the global efficiency within the neighborhood of node *i* and is closely related to the clustering coefficient.
7. The (weighted) clustering coefficient $\boldsymbol{F}_{cc}$ (Onnela et al., 2005) is defined as the average “intensity” (geometric mean) of all triangles associated with each node.
8. The node versatility $\boldsymbol{F}_{nv}$ (Shinn et al., 2017) assesses the consistency with which a node in a modular decomposition is linked to a particular module.

For the calculation of $\boldsymbol{F}_{pc}$ and $\boldsymbol{F}_{nv}$, which require module assignments, we employed a robust multi-resolution consensus community detection approach based on the Louvain algorithm (Blondel et al., 2008). This procedure was conducted as follows:

1. The Louvain algorithm was applied across 22 resolution parameters $\gamma =0.4\sim 2.5$ (step size 0.1). For each $\gamma$, $L=100$ independent iterations were performed to account for algorithmic stochasticity.
2. For each $\gamma$, a consensus matrix $\mathcal {C}(\gamma )$ was constructed, where its element $\mathcal {C}_{ij}(\gamma )$ represents the probability that nodes *i* and *j* belong to the same community across the 100 iterations: $\mathcal {C}_{ij}(\gamma ) = \frac{1}{L} \sum _{\ell =1}^{L} \mathbb {I}\left( c_i^{(\ell )}(\gamma ) = c_j^{(\ell )}(\gamma )\right)$, where $L=100$, $c_i^{(\ell )}(\gamma )$ is the community assignment of node *i* in the $\ell$-th run at resolution $\gamma$, and $\mathbb {I}(\cdot )$ is the indicator function;
3. To compute $\boldsymbol{F}_{pc}$, a final stable community partition was obtained by applying the Louvain algorithm (with $\gamma =1.0$) to the average consensus matrix $\bar{\mathcal {C}} = \frac{1}{|\Gamma |} \sum _{\gamma \in \Gamma } \mathcal {C}(\gamma )$. Here, $\Gamma$ denotes the set of resolution parameters, with $|\Gamma | = 22$.
4. Node versatility $\boldsymbol{F}_{nv}(i)$ was calculated by first computing a metric at each $\gamma$: $\boldsymbol{F}_i(\gamma )=\sum _j\sin (\pi \mathcal {C}_{ij}(\gamma ))/\sum _j \mathcal {C}_{ij}(\gamma )$. The final $\boldsymbol{F}_{nv}(i)$ is the average across all $\gamma$ values: $\boldsymbol{F}_{nv}(i) = \frac{1}{|\Gamma |} \sum _{\gamma \in \Gamma } \boldsymbol{F}_i(\gamma )$.

To analyze nodal topological properties in group networks, we employ the Signed Euclidean Distance (SED) to quantify discrepancies between CG and GG average networks. This comparison is performed for each nodal topological feature at various connection densities *p* in both MSN and MIND networks. Prior to SED computation, each nodal features from both groups of each connection density *p* are normalized to the [0, 1] range using Min-Max normalization:

9 $$\begin{aligned} \left[ \hat{\boldsymbol{F}}^C_\Phi (p), \hat{\boldsymbol{F}}^G_\Phi (p)\right] = \text {MinMax}_{\begin{array}{c} [0,1] \end{array}}\left( \left[ \boldsymbol{F}^C_\Phi (p), \boldsymbol{F}^G_\Phi (p)\right] \right) , \end{aligned}$$

where $\Phi \in \left\{ nd, ns, ec, pc, nc, le, nv\right\}$ denotes the nodal feature type. Here, $\boldsymbol{F}^C_\Phi$ and $\boldsymbol{F}^G_\Phi$ represent raw features of CG and GG average networks at connection density *p*, respectively, while $\hat{\boldsymbol{F}}^C_\Phi$ and $\hat{\boldsymbol{F}}^G_\Phi$ are their normalized counterparts. The SED is defined as:

10 $$\begin{aligned} D^u_\Phi (p) = {\text {sgn}}\left( \mu \left( \hat{\boldsymbol{F}}^G_\Phi (p)\right) - \mu \left( \hat{\boldsymbol{F}}^C_\Phi (p)\right) \right) \cdot \left\| \hat{\boldsymbol{F}}^G_\Phi (p) - \hat{\boldsymbol{F}}^C_\Phi (p) \right\| _2 \end{aligned}$$

The term $D^u_\Phi (p)$ quantifies the SED for feature $\Phi$ in brain network *u* ($u\in \left\{ MSN, MIND\right\}$).

To further examine hemispheric differences, we compute the mean SED between CG and GG using hemispheric subsets of each nodal feature vector:

11 $$\begin{aligned} \bar{D}^{L,u}_\Phi (p) = {\text {sgn}}\left( \mu \left( \hat{\boldsymbol{F}}^{L,G}_\Phi (p)\right) - \mu \left( \hat{\boldsymbol{F}}^{L,C}_\Phi (p)\right) \right) \cdot \frac{\left\| \hat{\boldsymbol{F}}^{L,G}_\Phi (p) - \hat{\boldsymbol{F}}^{L,C}_\Phi (p) \right\| _2}{N_L}, \end{aligned}$$

where $\hat{\boldsymbol{F}}^{L,G}_\Phi (p)$ and $\hat{\boldsymbol{F}}^{L,C}_\Phi (p)$ denote the first $N_L$ elements of $\hat{\boldsymbol{F}}^{G}_\Phi (p)$ and $\hat{\boldsymbol{F}}^{C}_\Phi (p)$, respectively, corresponding to the left hemisphere. A similar definition applies to $\bar{D}^{R,u}_\Phi (p)$ for the right hemisphere.

#### Individual Cognitive Analysis for MSNs and MIND Networks

This section examines how global brain network metrics relate to cognitive performance for both MSN and MIND. Seven typical global topological features (assortativity $f_a$, transitivity $f_t$, global efficiency $f_{ge}$, characteristic path length $f_{cpl}$, mean participation coefficient $f_{mpc}$, mean (global) weighted clustering coefficient $f_{mcc}$, mean versatility $f_{mv}$) and five hemispheric metrics (left-to-right intra-hemispheric ratio $R_{lr}$, intra-to-inter-hemispheric ratio $R_{ii}$, left intra-hemispheric strength $S_{LL}$, right intra-hemispheric strength $S_{RR}$ and inter-hemispheric strength $S_{LR}$) are analyzed.

1. Assortativity (Newman, 2002) $f_a$ is defined as the correlation coefficient for the degrees of neighboring nodes, which refers to the tendency of nodes in a network to link with other similar nodes.
2. Transitivity $f_t$ quantifies the ratio of closed to all possible triplets in a network, providing a global measure of segregation by capturing the extent of locally clustered connectivity (Rubinov & Sporns, 2010).
3. A network’s global efficiency (Latora & Marchiori, 2001) $f_{ge}$ is the reciprocal of the harmonic mean of its path lengths.
4. The characteristic path length (Watts et al., 1998) $f_{cpl}$ is the average shortest path length between all possible pairs of nodes in a network. Moreover, the characteristic path length of a network is strongly positively correlated with the network’s mean strength.
5. The mean participation coefficient can measure the global integration of a network (Solé-Casals et al., 2019), which is denoted as $f_{mpc}=\frac{1}{R} \sum \limits _{i \in R} \boldsymbol{F}_{pc}(i)$, where $R=308$, $\boldsymbol{F}_{pc}$ represents the participation coefficient vector of a network.
6. The mean clustering coefficient is denoted as $f_{mcc}=\frac{1}{R} \sum \limits _{i \in R} \boldsymbol{F}_{cc}(i)$, where $\boldsymbol{F}_{cc}$ represents the clustering coefficient vector of a network.
7. The mean versatility can measure the global integration of a network (Solé-Casals et al., 2019), which is denoted as $f_{mv}=\frac{1}{R} \sum \limits _{i \in R} \boldsymbol{F}_{v}(i)$, where $\boldsymbol{F}_{v}$ represents the versatility vector of a network.
8. The left intra-hemispheric strength $S_{LL}$, right intra-hemispheric strength $S_{RR}$ and inter-hemispheric strength $S_{LR}$ are defined in Eq. 8.
9. The left-to-right intra-hemispheric ratio is calculated as $R_{lr}=\frac{S_{LL}}{S_{RR}}$.
10. The intra-to-inter-hemispheric ratio is denoted as $R_{ii}=\frac{S_{LL} +S_{RR}}{S_{LR}}$.

We assess the relationship between cognitive performance and global metrics of individual brain networks using **Spearman correlation coefficients (SCCs)**. SCC is a non-parametric measure of rank correlation, evaluating the statistical dependence between the rankings of two variables. It determines how well the relationship between two variables can be described using a monotonic function. The SCC, denoted as $\rho$, ranges from -1 to 1: $\rho =1$ signifies a perfect positive monotonic relationship; $\rho =-1$ signifies a perfect negative monotonic relationship; $\rho =0$ indicates no monotonic relationship.

In this work, given the small sample size ($N = 29$) and the exploratory nature of the study, we interpret $\rho> 0.2$ as indicating a potentially meaningful monotonic association, corresponding approximately to a small-to-moderate effect, rather than as a formal threshold for statistical significance.

For this analysis, we define $\rho ^p_{u}(\boldsymbol{\phi }^p,\boldsymbol{C}_{IQ})$ to represent the SCC between a global metric $\boldsymbol{\phi }^p$ of networks with connection density *p* and IQ scores $\boldsymbol{C}_{IQ}$, where $u \in \left\{ MSN, MIND\right\}$ denotes different brain networks, $\boldsymbol{\phi } \in \{\boldsymbol{f}_a, \boldsymbol{f}_t, \boldsymbol{f}_{ge}, \boldsymbol{f}_{cpl}, \boldsymbol{f}_{mpc},$ $\boldsymbol{f}_{mcc}, \boldsymbol{f}_{mv}, \boldsymbol{S}_{LL}, \boldsymbol{S}_{RR}, \boldsymbol{S}_{LR}, \boldsymbol{R}_{lr}, \boldsymbol{R}_{ii} \}$ represents various global metric vectors of all subjects.

To further assess group differences across each global metric, we apply the **Mann-Whitney** *U* **test**, a non-parametric test used to determine whether there is a significant difference between the distributions of two independent groups. This test is particularly useful when the assumptions of normality are not met. The Mann-Whitney *U* test is defined as follows:

12 $$\begin{aligned} P_p^{\boldsymbol{\phi }}=U(\boldsymbol{\phi }_p^G, \boldsymbol{\phi }_p^C) \end{aligned}$$

where $\boldsymbol{\phi }_p^G$ and $\boldsymbol{\phi }_p^C$ represent the metric vectors for the GG and CG groups, respectively, at density *p*. The resulting $P_p^{\boldsymbol{\phi }}$ is the probability of the difference between the two groups for each global metric, with lower values (typically $P_p^{\boldsymbol{\phi }} \le 0.05$) indicating statistical significance. Specifically, we computed these measures for the GG and CG groups at a connection density of $p = 0.1$.

Furthermore, to account for multiple comparisons and control the false discovery rate (FDR), we applied the Benjamini-Hochberg FDR correction (Benjamini & Hochberg, 1995) to the 12 $P_p^{\boldsymbol{\phi }}$ values derived from the 12 global metrics ($\boldsymbol{\phi }$) at connection density $p = 0.1$. This correction was performed separately for the MSN and MIND networks. The FDR-adjusted $P_p^{\boldsymbol{\phi }}$ values are denoted as $P_{FDR}^{\boldsymbol{\phi }}$.

## Results

### Group Analysis Results of MSNs and MIND Networks

**Fig. 3:**
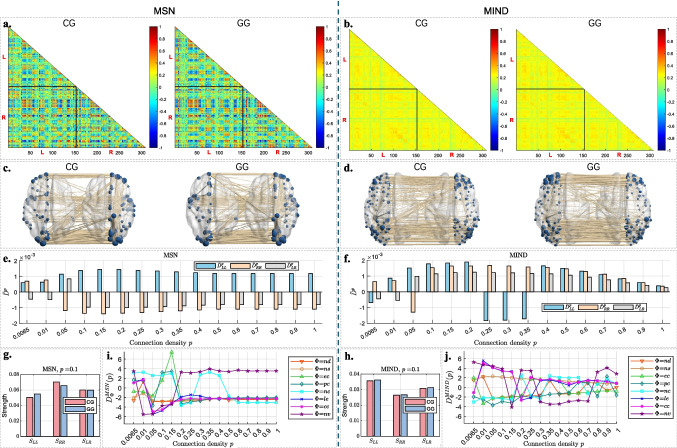
Comparative analysis of brain networks between CG and GG for both MSN (left part) and MIND (right part): **a** and **b** show heatmaps of group-average networks for CG and GG, organized by hemispheric node order, for MSN (**a**) and MIND (**b**), respectively; color values range from -1 to 1, with warmer colors representing higher values. **c** and **d** are visualization of the top 1% strongest connections in CG and GG average networks for MSN (**c**) and MIND (**d**); edge thickness represents connection weight, node size reflects degree. **e** and **f** display the mean SED of hemispheric connections ($\bar{D}_{LL}$, $\bar{D}_{RR}$, $\bar{D}_{LR}$) between two groups across different connection densities for MSN (**e**) and MIND (**f**). **g** and **h** show the hemispheric connection strengths ($S_{LL}$, $S_{RR}$ and $S_{LR}$) at connection density $p=0.1$ for MSN (**g**) and MIND (**h**). **i** and **j** present SED ($D^u_\Phi (p)$, where $\Phi \in \left\{ nd, ns, ec, pc, nc, le, nv\right\}$, $u\in \left\{ MSN, MIND\right\}$) of each nodal topological feature between CG and GG average networks on across different connection densities *p* for MSN (**i**) and MIND (**j**). Features include node degree ($\Phi =nd$), node strength ($\Phi =ns$), eigenvector centrality ($\Phi =ec$), participation coefficient ($\Phi =pc$), node (betweenness) centrality ($\Phi =nc$), local efficiency ($\Phi =le$), clustering coefficient ($\Phi =cc$) and node versatility ($\Phi =nv$)

As described in Section 2.2.2, we performed group analyses for MSNs and MIND networks to investigate structural differences between CG and GG. The results are presented as follows: Fig. 3 illustrates the comparison of CG and GG in terms of group average brain networks and hemispheric connections for MSN and MIND.

#### Hemispheric Analysis for Group Average Brain Networks

- **Heatmap comparison for group average brain networks.**

Figure 3**a** and 3**b** show heatmaps of CG (left) and GG (right) average networks organized by left and right hemispheric node order for MSN (**a**) and MIND (**b**). The heatmaps reflect the fact that MSNs encompass both positive and negative values ranging from $-1$ to 1, whereas MIND networks only consist of positive values ranging from 0 to 0.5. However, it appears that there is no discernible visual distinction between the CG and GG in the heatmaps of both the group average MSNs and MIND networks.

- **Comparison of top 1% group brain networks.**

Figure 3**c** and **d** display the top 1% of CG (left) and GG (right) average brain networks for MSN (**c**) and MIND (**d**), visualized using BrainNet Viewer (Xia et al., 2013). These networks are based on the heatmaps shown in **a** and **b**, respectively. For a brain network with $R=308$ nodes, the top 1% includes $[(R-1)R/2]\times 1\%=474$ connections. The visualization shows that connections are more localized in group average MSNs, while connections are more evenly distributed across the group average MIND networks. Moreover, the top 1% connections in group average MSNs display clearer distinctions between CG and GG networks than those in the MIND average networks.

- **Intra-/Inter-hemispheric analysis for group average brain networks.**

Figure 3**e** and 3**f** display the mean SED of three types of hemispheric connections ($\bar{D}_{LL}$, $\bar{D}_{RR}$ and $\bar{D}_{LR}$) between the two groups’ average networks across different connection densities *p* for MSN and MIND, respectively. It can be observed that:

- For both MSN and MIND, the magnitudes of intra-hemispheric mean Euclidean distances ($|\bar{D}^p_{LL}|$ and $|\bar{D}^p_{RR}|$) consistently exceed those of inter-hemispheric distances ($|\bar{D}^p_{LR}|$) across all connection densities. Specifically, the largest mean Euclidean distances ($|\bar{D}^p_{LL}|$, $|\bar{D}^p_{RR}|$, and $|\bar{D}^p_{LR}|$) in MIND are slightly higher than those in MSN for each corresponding variable. Notably, all three types of hemispheric distances show higher magnitudes within the density range of $0.05 \le p \le 0.4$.
- For MSN, $\bar{D}^p_{LL}$ remains positive, while both $\bar{D}^p_{RR}$ and $\bar{D}^p_{LR}$ are negative throughout the range $0.05 \le p \le 0.4$. This suggests that the GG average MSN exhibits stronger left intra-hemispheric connections, but weaker right intra-hemispheric and inter-hemispheric connections compared to CG.
- For MIND, $\bar{D}^p_{LL}$, $\bar{D}^p_{RR}$ and $\bar{D}^p_{LR}$ fluctuate around zero across different connection densities, suggesting inconsistent average (strength) differences between groups in intra-/inter-hemispheric connections.

For further exploration, Fig. 3**g** and 3**h** illustrate the hemispheric connection strengths ($S_{LL}$, $S_{RR}$, and $S_{LR}$) at a representative density of $p = 0.1$.

- For MSN, both CG and GG exhibit stronger $S_{RR}$ than $S_{LL}$. However, compared to CG, GG exhibits stronger $S_{LL}$, but weaker $S_{RR}$ and slightly weaker $S_{LR}$, aligning with the observed pattern of $\bar{D}^p_{LL}$, $\bar{D}^p_{RR}$ and $\bar{D}^p_{LR}$ in Fig. 3**e**.
- In contrast, for MIND, both CG and GG exhibit stronger $S_{LL}$ than $S_{RR}$, and stronger $S_{LR}$ than $S_{RR}$. However, the differences between CG and GG in $S_{LL}$, $S_{RR}$ and $S_{LR}$ are minimal, which is consistent with the fluctuating signs of $\bar{D}^p_{LL}$, $\bar{D}^p_{RR}$ and $\bar{D}^p_{LR}$, as shown in Fig. 3**f**.

Overall, these hemispheric results suggest that MSN captures more consistent and interpretable group differences than MIND within the range $p = 0.05\sim 0.4$. In this density range, higher cognitive performance in the gifted group appears to be associated with relatively stronger left intra-hemispheric connectivity and weaker inter-hemispheric connectivity, whereas MIND shows less stable and less clearly interpretable hemispheric patterns.

#### Analysis Results of Nodal Topological Features in Group Networks

- **Group-wise SED of nodal topological features across different connection densities.**

Figure 3**i** and 3**j** present the SED $\boldsymbol{D}_{\Phi }(p)$ between the CG and GG average networks on each nodal topological feature $\Phi$ at different connection densities *p* for MSNs and MIND networks, respectively. Each feature is represented by a distinct color and symbol, allowing for visual comparison across connection densities. It can be observed that:

- The values of $D_{nd}$ (node degree, red down-pointing triangles), $D_{ns}$ (node strength, brown circles), $D_{ec}$ (eigenvector centrality, green triangles) and $D_{nc}$ (node betweenness centrality, cyan tangles) show relatively small Euclidean distances between the two groups across most *p* values in both MSN and MIND, suggesting these metrics have limited effectiveness in cognitive analysis.
- The trends of $D_{cc}$ (clustering coefficient, magenta stars) and $D_{le}$ (local efficiency, blue crosses) exhibit similar patterns as *p* varies in both MSN and MIND. For MSN, $D_{cc}$ and $D_{le}$ show relatively high Euclidean distances with negative signs as $0.05 \le p\le 0.15$. On the other hand, for MIND, both metrics show relatively high Euclidean distances with positive signs as $0.01 \le p\le 0.15$. These observations suggest that both clustering coefficient and local efficiency are effective metrics for evaluating the two groups’ distance in both MSN and MIND. However, GG shows lower mean clustering coefficient and mean local efficiency compared to that of CG across different connection densities in MSN, while showing the opposite in MIND.
- The values of $D_{pc}$ (participation coefficient, teal diamonds) and $D_{nv}$ (node versatility, purple stars) exhibit relatively large Euclidean distances between the two groups as $p \le 0.15$ for both MSN and MIND. This suggests that both metrics are effective in evaluating the group distance within this connection density range. The fluctuating signs indicate that the mean participation coefficient and node versatility of the group networks may be unstable in cognitive analysis for both MSN ($p = 0.05 \sim 0.2$) and MIND ($p = 0.5 \sim 1$). Notably, the negative $D_{pc}$ in MIND for $p \le 0.5$ indicates that GG shows a lower mean participation coefficient compared to CG in MIND.

- **Hemispheric analysis on the nodal topological features between groups.**

**Fig. 4:**
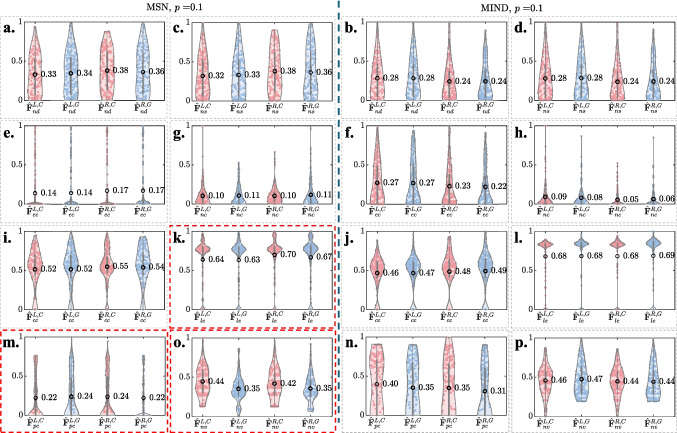
Left and right hemispheric nodal features ($\hat{\boldsymbol{F}}^{L,C}_\Phi$, $\hat{\boldsymbol{F}}^{L,G}_\Phi$, $\hat{\boldsymbol{F}}^{R,C}_\Phi$, and $\hat{\boldsymbol{F}}^{R,G}_\Phi$, where $\Phi \in \{nd, ns, ec, pc, nc, le, nv\}$) for CG (red) and GG (blue) across MSN (left) and MIND (right) at connection density $p = 0.1$. Values inside the violins represent the mean of each feature. **a** and **b**: $\Phi = nd$ (node degree); **c** and **d**: $\Phi = ns$ (node strength); **e** and **f**: $\Phi = ec$ (eigenvector centrality); **g** and **h**: $\Phi = nc$ (node betweenness centrality); **i** and **j**: $\Phi = cc$ (clustering coefficient); **k** and **l**: $\Phi = le$ (local efficiency); **m** and **n**: $\Phi = pc$ (participation coefficient); **o** and **p**: $\Phi = nv$ (node versatility)

**Fig. 5:**
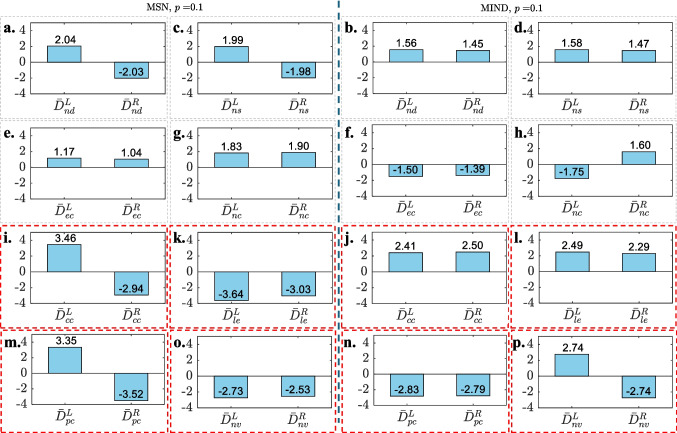
Mean SEDs of hemispheric nodal features ($\bar{D}^{L,u}_\Phi (p), \bar{D}^{R,u}_\Phi (p)$, where $\Phi \in \{nd, ns, ec, pc, nc, le, nv\}$, $u\in \left\{ MSN, MIND\right\}$) between CG and GG at connection density $p = 0.1$ for MSN (left) and MIND (right). The subfigure order of nodal features is the same as that in Fig. 4

To further examine hemispheric differences in nodal topological features between CG and GG, violin plots in Fig. 4 present distributions of the normalized left- and right-hemispheric nodal features ($\hat{\boldsymbol{F}}^{L,C}_\Phi (p)$, $\hat{\boldsymbol{F}}^{L,G}_\Phi (p)$, $\hat{\boldsymbol{F}}^{R,C}_\Phi (p)$, and $\hat{\boldsymbol{F}}^{R,G}_\Phi (p)$) and boxes in Fig. 5 presents the mean SED of these hemispheric features between the two groups at a representative connection density of $p = 0.1$, for both MSN (left) and MIND (right) networks.

From Figs. 4 and 5, we observe the following:

- Hemispheric nodal degree (subfigure **a** and **b**), nodal strength (subfigure **c** and **d**), eigenvector centrality (subfigure **e** and **f**), and node betweenness centrality (subfigure **g** and **h**) show minimal distribution and average differences (in Fig. 4) between CG and GG, with relatively small mean SEDs (in Fig. 5) across both MSN and MIND. These features exhibit similar patterns in both hemispheres and groups, suggesting that hemispheric and group-specific differences in these topological features are minimal.
- Clustering coefficient shows little average differences between the two groups (Fig. 4**i** and 4**j**), but visual distribution differences are noticeable in MSN (Fig. 4**i**). Local efficiency shows a notable lower average in GG than CG for the right hemisphere in MSN (Fig. 4**k**), but minimal differences in MIND (Fig. 4**l**). Both features display relatively higher Euclidean distances (Fig. 5**k** and 5**l**), particularly in MSN, suggesting more prominent hemispheric differences in these features for the MSN network. While the trends of $D_{cc}$ and $D_{le}$ in Fig. 3**i** and 3**j** exhibit similar patterns as *p* varies in both MSN and MIND, the distributions of these two nodal feature values are notably different.
- The participation coefficient shows notable differences in distribution between the two groups across both hemispheres for both MSN (Fig. 4**m**) and MIND (Fig. 4**n**). Specifically, in the MSN network, GG exhibits a higher average in the left hemisphere compared to CG, while the reverse trend is observed in the MIND network. In the right hemisphere, GG has a lower average than CG for both MSN and MIND networks. Additionally, the participation coefficient demonstrates relatively high Euclidean distances in both hemispheres for both MSN (Fig. 5**m**) and MIND (Fig. 5**n**).
- Node versatility reveals visual distribution differences for both MSN (Fig. 4**o**) and MIND (Fig. 4**p**). Specifically, for MSN, the averages for both hemispheres are notably lower in GG than CG, whereas MIND shows minimal differences. Node versatility shows relatively high Euclidean distances in both MSN (5**o**) and MIND (5**p**).

These observations align with the results in Fig. 3**i** and 3**j**, where the clustering coefficient, local efficiency, participation coefficient and node versatility of group average networks show higher Euclidean distances between CG and GG for both MSN and MIND.

Taken together, the nodal analyses show that simple connectivity metrics (degree, strength, betweenness, eigenvector centrality) carry limited information about group differences, whereas more advanced segregation and integration metrics (clustering coefficient, local efficiency, participation coefficient, and node versatility) are more sensitive to cognitive status. These effects are particularly clear in MSNs and are most pronounced within the density range $p = 0.05\sim 0.15$, further supporting this window as a useful operating range for structural network studies in our cohort.

### Results of Individual Cognitive Analysis for MSNs and MIND Networks

This section presents the results of individual-level cognitive analyses based on global metrics for both MSNs and MIND networks. We first examine the SCCs between IQ scores and the global metrics (seven global topological features and five hemispheric metrics). Regression plots are provided to visualize these relationships at connection density $p = 0.1$. Furthermore, we report group differences between GG and CG groups for each global metric using Mann-Whitney *U* tests at a connection density of $p = 0.1$, with results presented as both uncorrected ($P_p^{\boldsymbol{\phi }}$) and FDR-adjusted ($P_{FDR}^{\boldsymbol{\phi }}$) values.

#### The Relationship Between IQ and Global Topological Features

**Fig. 6:**
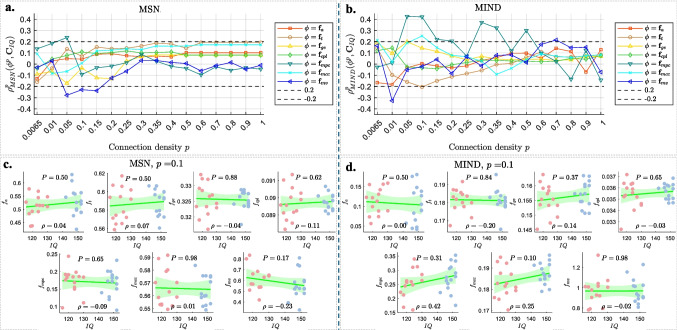
The SCC results between IQ and various global topological features ($\boldsymbol{\phi } \in \left\{ \boldsymbol{f}_a, \boldsymbol{f}_t, \boldsymbol{f}_{ge}, \boldsymbol{f}_{cpl}, \boldsymbol{f}_{mpc}, \boldsymbol{f}_{mcc}, \boldsymbol{f}_{mv} \right\}$) of brain networks for MSN (left) and MIND (right). **a** and **b** display the SCCs $\rho ^p_{u}(\boldsymbol{\phi }^p,\boldsymbol{C}_{IQ})$ across varying connection densities *p* (x-axis). **c** and **d** present scatter plots at a connection density of $p=0.1$, with IQ scores (x-axis) plotted against various global topological features (y-axis). Each scatter plot includes data points (light red: CG; light blue: GG), regression lines, shaded confidence intervals, and the corresponding $P^{\boldsymbol{\phi }}$ values at the top and SCC $\rho$ values at the bottom of each subplot

**Table 2:** Individual cognitive analysis results for MSN and MIND networks: SCCs ($\rho$) between IQ scores and 12 global metrics, and Mann-Whitney *U* test *P*-values (uncorrected $P^{\phi }$ and FDR-corrected $P^{\phi }_{FDR}$) for group differences (GG vs. GC) at connection density $p=0.1$. Bold $\rho$ values indicate $|\rho |>0.2$; bold $P^{\boldsymbol{\phi }}$ values indicate $P^{\boldsymbol{\phi }}<0.05$

	MSN			MIND		
$\boldsymbol{\phi }$	$\rho _{MSN}(\boldsymbol{\phi },\boldsymbol{C}_{IQ})$	$P^{\phi }$	$P^{\phi }_{FDR}$	$\rho _{MIND}(\boldsymbol{\phi },\boldsymbol{C}_{IQ})$	$P^{\boldsymbol{\phi }}$	$P^{\boldsymbol{\phi }}_{FDR}$
$\boldsymbol{f}_a$	0.04	0.50	0.85	0.00	0.50	1.00
$\boldsymbol{f}_t$	0.07	0.50	0.85	**-0.20**	0.84	1.00
$\boldsymbol{f}_{ge}$	-0.04	0.88	0.96	0.14	0.37	1.00
$\boldsymbol{f}_{cpl}$	0.11	0.62	0.85	-0.03	0.65	1.00
$\boldsymbol{f}_{mpc}$	-0.09	0.65	0.85	**0.42**	0.31	1.00
$\boldsymbol{f}_{mcc}$	0.01	0.98	0.98	**0.25**	0.10	1.00
$\boldsymbol{f}_{mv}$	**-0.23**	0.17	0.51	-0.02	0.98	1.00
$\boldsymbol{S}_{LL}$	**0.29**	0.16	0.51	0.08	0.68	1.00
$\boldsymbol{S}_{RR}$	-0.01	0.71	0.85	0.01	0.88	1.00
$\boldsymbol{S}_{LR}$	**-0.22**	0.12	0.51	-0.02	0.68	1.00
$\boldsymbol{R}_{lr}$	**0.20**	0.42	0.85	0.00	1.00	1.00
$\boldsymbol{R}_{ii}$	**0.34**	**0.02**	0.26	0.17	0.74	1.00

As described in 2.2.3, we analyzed the Spearman correlations between IQ scores and the seven global topological features of brain networks across different connection densities *p*. The overall correlation trends are displayed in Fig. 6**a** (MSN) and 6**b** (MIND). Detailed scatter plots at a representative density of $p=0.1$ are provided in Fig. 6**c** and **d**, respectively.

From Fig. 6, we can observe the following:

- Assortativity $f_a$ (red tangles) exhibits relatively low correlations with IQ across all *p* values for MSN, consistently showing positive correlations ($\rho \lesssim 0.1$) when $p \ge 0.01$. For MIND, $f_a$ shows slightly stronger negative correlations ($\rho \gtrsim -0.2$) when $p \le 0.01$.
- Transitivity $f_t$ (brown circles) exhibits positive correlations with IQ when $p \ge 0.05$ for MSN, with slightly higher positive correlations ($\rho \lesssim 0.2$) as $p \ge 0.15$. However, for MIND, $f_t$ shows negative correlations with IQ in the range of $0.01 \le p \le 0.3$, with stronger negative correlations ($\rho = -0.2$ as shown in the second panel of Fig. 6**d**) observed in the range $0.05 \le p \le 0.15$.
- Global efficiency $f_{ge}$ (yellow triangles) shows negative correlations with IQ when $p \le 0.2$, reaching the strongest correlation ($\rho \gtrsim 0.2$) at $p = 0.05$. In contrast, $f_{ge}$ shows positive correlations with IQ across all *p* values in MIND, reaching the strongest correlation ($\rho = 0.2$) at $p = 0.05$.
- Characteristic path length $f_{cpl}$ (green diamonds) exhibits low correlations with IQ across *p* values for both MSN and MIND.
- Correlations between IQ and the mean participation coefficient $f_{mpc}$ (teal triangles) exhibit sharp fluctuations as the connection density *p* changes for both MSN and MIND. The instability of $f_{mpc}$ is consistent with previous group analysis results in Fig. 3**i** and 3**j**. Although $f_{mpc}$ shows significant correlations for $0.05\le p\le 0.15$ (e.g., $\rho = 0.42$ when $p = 0.1$ in MIND, as shown in the fifth panel of Fig. 6**d**), the positive correlations in MIND appear inconsistent with the group-level result, which shows a lower mean participation coefficient in GG compared to CG.
- The mean weighted clustering coefficient $f_{mcc}$ (cyan crosses) shows low correlations with IQ across *p* values in MSN, while it shows a significantly positive correlation with IQ in the range of $0.05 \le p \le 0.1$ in MIND (e.g., $\rho =0.25$ when $p=0.1$ as shown in the sixth panel of Fig. 6**d**).
- Mean versatility $f_{mv}$ (blue triangles) shows a significantly negative correlation with IQ for $0.05 \le p \le 0.15$ in MSN (e.g., $\rho =-0.23$ when $p=0.1$ as shown in the last panel of Fig. 6**c**), while in MIND, it exhibits sign fluctuations across different *p* values.

Additionally, Table 2 presents SCCs ($\rho$) between IQ scores and each global metric, and the Mann-Whitney *U* test *P*-values (uncorrected $P^{\phi }$ and FDR-corrected $P^{\phi }_{FDR}$) across each global metric for group differences (GG vs. GC) at connection density $p=0.1$. From Table 2, it can be observed that stronger correlations generally tend to have smaller $P^\phi$ values in more cases. Among the seven global topological features, none show significant group differences. Notably, MSN shows smaller *P*-values for most global metrics compared to MIND, especially after FDR correction, suggesting that group differences, while non-significant, are more discernible using MSN.

In summary, global topological metrics show network-specific and density-dependent associations with IQ. MSNs tend to yield more stable and interpretable correlations with cognitive performance, particularly for mean versatility, and align more consistently with group-level analysis. In contrast, MIND networks often display sign reversals or weak effects across densities. Most meaningful associations emerge within $p = 0.05\sim 0.15$, reinforcing this range as a practical trade-off between sparsity, stability, and cognitive sensitivity.

#### The Relationship Between IQ and Global Hemispheric Metrics

**Fig. 7:**
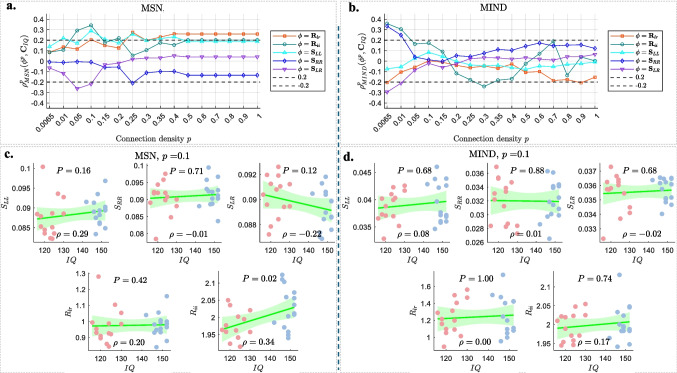
The SCC results illustrating the relationships between IQ and various global hemispheric metrics ($\boldsymbol{\phi } \in \left\{ \boldsymbol{S}_{LL}, \boldsymbol{S}_{RR}, \boldsymbol{S}_{LR}, \boldsymbol{R}_{lr}, \boldsymbol{R}_{ii} \right\}$) of brain networks for MSN (left) and MIND (right). **a** and **b** display the SCCs $\rho ^p_{u}(\boldsymbol{\phi }^p,\boldsymbol{C}_{IQ})$ across varying connection densities *p* (x-axis). **c** and **d** present scatter plots at $p=0.1$, with IQ scores (x-axis) vs. hemispheric metrics (y-axis), including data points (CG in light red, GG in light blue), regression lines, confidence intervals, and the corresponding $P^{\boldsymbol{\phi }}$ values at the top and SCC $\rho$ values at the bottom of each subplot

As described in 2.2.3, we examined the Spearman correlations between IQ scores and the five hemispheric metrics of brain networks across different connection densities *p*. The results are illustrated in Fig. 7**a** (for MSN) and 7**b** (for MIND). Additionally, scatter plots of IQ scores versus each hemispheric metric at a connection density of $p = 0.1$ are presented in Fig. 7**c** (for MSN) and 7**d** (for MIND).

For MSN, the following observations can be made:

- IQ is consistently positively correlated with the ratio of left to right intra-hemispheric connections $R_{lr}$, the intra-to-inter-hemispheric ratio $R_{ii}$, and the left hemispheric strength $S_{LL}$ across all values of *p*. Notably, these correlations are stronger in the range $0.05 \le p \le 0.15$, with $\rho = 0.29$ for $S_{LL}$ at $p = 0.1$ (Fig. 7**c**, first panel), $\rho = 0.20$ for $R_{lr}$ at $p = 0.1$ (Fig. 7**c**, fourth panel), and $\rho = 0.34$ for $R_{ii}$ at $p = 0.1$ (Fig. 7**c**, last panel).
- IQ shows negative correlations with inter-hemispheric strength $S_{LR}$ in the range $0.05\le p \le 0.1$, with $\rho =-0.22$ at $p=0.1$ (Fig. 7**c**, first panel).
- Correlations between IQ and right intra-hemispheric strength ($S_{RR}$) are minimal for most *p* values, except at $p = 0.25$, where $\rho \lesssim -0.2$.

In contrast, for MIND, the correlations between IQ and various hemispheric metrics are generally weak and unstable, only showing brief significance at very low or high connection densities:

- IQ shows low correlations with $R_{lr}$ for most *p* values, except at $p = 0.0065$ and $p \ge 0.7$, where $\rho \simeq -0.2$.
- The correlations between IQ and $R_{ii}$ are inconsistent: positive correlations for $p\le 0.1$ and at $p=0.7$, but negative correlations for $0.25\le p\le 0.4$ and at $p=0.8$.
- IQ shows very weak correlations with $S_{LL}$ across all *p* values.
- IQ generally correlates weakly with $S_{RR}$ and $S_{LR}$, except at very low densities $p\le 0.01$, where $\rho> 0.2$ for $S_{RR}$ and $\rho < -0.2$ for $S_{LR}$.

Finally, we directly compared the sensitivity of MSN and MIND in detecting group differences (GG vs. CG) in these hemispheric metrics at $p=0.1$. As summarized in Table 2, the intra-to-inter-hemispheric ratio ($R_{ii}$) showed a nominally significant group difference ($P^{\phi } < 0.05$) for MSN, though this did not survive FDR correction ($P^{\phi }_{FDR}> 0.05$). Notably, across all five metrics, the *P*-values derived from MSN networks were consistently smaller than those from MIND networks, both before and after FDR correction.

Overall, the hemispheric metrics highlight a clearer and more coherent relationship between structural organization and IQ in MSN than in MIND. In MSN, higher IQ is tentatively associated with stronger left intra-hemispheric connectivity, reduced inter-hemispheric strength, and an increased intra-to-inter-hemispheric ratio, particularly for $p = 0.05\sim 0.15$. By contrast, MIND metrics show weaker and less stable associations with IQ, suggesting that MSN may be more sensitive to hemispheric patterns relevant for cognitive performance in this sample.

## Discussion

This study explored the structural differences between MSNs and MIND networks, focusing on connectivity and topology patterns derived from sMRI scans and cognitive test data from 29 male children. The analyses at both group and individual levels revealed several potentially important findings regarding the impact of connection density, hemispheric distinctions, and the relationship between brain network features and cognitive performance. These results provide preliminary evidence for the distinct characteristics of MSNs and MIND networks and offer insights into their implications for understanding cognitive abilities.

- **The construction of MSN and MIND network.**

Although MSNs and MIND networks are built with the same 5 types of morphometric features, they differ significantly in data representation and computational methods. MSNs (Seidlitz et al., 2018) capture the overall structural connectivity through Pearson’s correlation of region-level aggregates, resulting in edge values that range from -1 to 1. This construction emphasizes macroscopic structural similarities between regions and provides a higher-level understanding of brain network organization. In contrast, MIND networks (Sebenius et al., 2023) are based on the distribution of MRI vertex features, utilizing symmetric KL divergence, which captures distributional similarities across regions, with edge values ranging from 0 to 0.5. This methodological difference results in distinct topological characteristics between the two types of networks. Additionally, in our cohort, MSN appeared to provide a more robust and reliable framework for analyzing structural brain-cognition relationships. This could partially explain why many MSN methods are reported in recent studies (Li et al., 2021; Vuksanović, 2022; Sun et al., 2024b, a; Janssen et al., 2025). However, Sebenius et al. (2023) found MIND network phenotypes to be more reliable, with gene co-expression between cortical areas more strongly linked to MIND networks than to MSNs. Nonetheless, as discussed in a later section “The relationship between topological features and cognitive performance”, the two network types still exhibit certain consistent patterns in group-level topological analyses, which may result from using the same types of morphometric features.

- **The effects of connection density.**

Varying connection densities provide an important perspective on how different levels of connectivity shape overall network organization and their associations with cognitive abilities. In this study, connection density was found to significantly influence both the structural properties of brain networks and the relationships between these properties and cognitive performance in both MSN and MIND.

- In group comparisons, both MSN and MIND exhibited higher Euclidean distances between CG and GG in left/right intra-hemispheric and inter-hemispheric connections within the density range $0.05 \le p \le 0.4$. Moreover, nodal topological features—such as local efficiency, clustering coefficient, participation coefficient, and node versatility—showed greater separation between groups within $0.05\le p\le 0.15$ for MSN, $0.01\le p\le 0.15$ for MIND.
- At the individual level, MSN showed relatively stable correlations and fewer reversals in sign across *p* values, and displayed stronger correlations with global topological properties (e.g., mean versatility) and hemispheric metrics–such as the intra-to-inter-hemispheric ratio ($R_{ii}$) and left intra-hemispheric strength ($S_{LL}$)–within $0.05 \le p \le 0.15$. In contrast, MIND showed more variable correlation patterns: stronger correlations with global features within $0.05 \le p \le 0.15$ (e.g., transitivity and mean clustering coefficient) and at $p \le 0.01$ (e.g., assortativity), as well as stronger correlations with certain hemispheric metrics (e.g., right intra-hemispheric strength and inter-hemispheric strength) only at very low densities ($p \le 0.01$).

Based on these observations, we tentatively recommend a connection density range of $p = 0.05 \sim 0.15$ for cognitive network studies using networks of comparable scale. This range appears to preserve potentially biologically robust connections, highlighting the core architecture of brain networks. Moreover, it was associated with stable and interpretable results, relatively maximized inter-group differences in connectivity and nodal topology, and potentially enhanced the reliability of structure-cognition relationships in our analysis. At lower densities ($p \le 0.05$), the values of the analyzed metrics are generally low or unstable with sharp fluctuations. Although sporadically notable correlations with cognitive performance may occur, such patterns are not reliable. This instability likely stems from excessive sparsification, which drastically alters network topology, distorts meaningful architectural properties, and fails to represent the original network organization. At higher densities ($p \ge 0.15$), statistical effects diminish and computational efficiency may be adversely affected. The recommended connection density range contrasts with certain studies, such as Sebenius et al. (2023), which employed very low connection densities ($p = 0.01$). However, it aligns with other works, including (Seidlitz et al., 2018; Solé-Casals et al., 2019), where a connection density of $p = 0.1$ was effectively used. Further research with larger samples is needed to confirm the optimal density range across different populations and network construction methods.

- **The relationship between topological features and cognitive performance.**

Our analysis revealed distinct nodal and global topological profiles for MSN and MIND networks, each of which was associated with cognitive performance in contrasting ways. This study examined how these relationships varied with connection density, though some metrics (like the mean participation coefficient) proved unstable. Therefore, the following discussion mainly focuses on the recommended range of connection density $p = 0.05 \sim 0.15$. Future work could employ the area-under-the-curve (AUC) approach (Hosseini et al., 2012) across densities to derive more stable composite measures of these network-cognition associations.

(1) *Nodal topological features.* The analysis of nodal topological features revealed a clear hierarchy of sensitivity to cognitive differences. Basic connectivity measures, including nodal degree, nodal strength, eigenvector centrality, and node betweenness centrality, demonstrated minimal group differences and limited relevance with cognitive performance. In contrast, advanced segregation metrics (clustering coefficient and local efficiency, reflecting specialized processing within modules) and advanced integration metrics (participation coefficient and node versatility, reflecting global information exchange across modules) emerged as the most sensitive indicators, showing consistently elevated Euclidean distances between groups across both network types. The hemispheric analysis of the nodal topological features further refined this understanding. These results suggest that cognitive performance might be more strongly associated with higher-order network properties–specifically, the capacity for both modular segregation and global integration–than with simple connection strength or density.

These findings appear consistent with previous studies. Janssen et al. (2025) reported that reduced morphometric similarity within the default mode network (a functional module) is related to cognitive impairment, while our findings highlight that enhanced modular integration is associated with better cognitive performance. Furthermore, Vuksanović (2022) reported that modular metrics, such as flexibility in a multilayer MSN, are associated with age and IQ. However, that study did not reveal hemispheric differences in modular organization, particularly in hub distribution. In contrast, both MSN and MIND analyses in our work demonstrate clear hemispheric asymmetries related to cognitive performance in the participation coefficient, a key metric for identifying and classifying hub nodes.

(2) *Global topological features.* At the individual level, analysis of global topological features revealed fundamentally distinct relationships with cognitive performance between MSN and MIND networks.

- Both **assortativity** ($f_a$) and **characteristic path length** ($f_{cpl}$) showed minimal correlations with IQ in both network types. The latter finding is consistent with the group-level analysis, which also revealed negligible Euclidean distances in nodal strength between groups, suggesting that these metrics may show limited sensitivity to cognitive performance in SBN analysis.
- **Mean participation coefficient** ($f_{mpc}$) exhibited pronounced fluctuations in the magnitude and direction of its correlation with IQ across connection densities in both MSN and MIND, despite occasionally high SCC values. This instability aligns with the variable inter-group SEDs observed in participation coefficient across densities. Additionally, the correlations directions in individual analysis appear inconsistent with the group-level results. These observations may render $f_{mpc}$ an unreliable correlate of cognitive ability. The instability of the mean participation coefficient ($f_{mpc}$) likely stems from the combined effect of the stochastic Louvain algorithm (Lancichinetti & Fortunato, 2009) and its interaction with graph topology defined by connection density (*p*).
- **Transitivity** ($f_t$) showed a slight positive correlation with IQ in MSN but a negative correlation in MIND.
- **Global efficiency** ($f_{ge}$) demonstrated a slight negative correlation with IQ in MSN, contrasting with a positive correlation in MIND. This pattern of opposing relationships aligns with the SEDs observed for local efficiency in the group-level analysis, which were negative for MSN and positive for MIND, suggesting a consistent divergence in how efficiency metrics relate to cognitive performance across network types.
- **Mean clustering coefficient** ($f_{mcc}$) was weakly correlated with IQ in MSN but showed a positive correlation in MIND. The positive correlation in MIND aligns with the positive SEDs observed for the clustering coefficient in the group-level analysis. In contrast, the weak correlation in MSN does not align with the negative SEDs found in the group-level analysis for this network type.
- **Mean versatility** ($f_{mv}$) showed no strong or consistent relationship with IQ in MIND networks across connection densities—except at an isolated low density ($p = 0.01$)—and exhibited considerable fluctuations, aligning with group-level analyses where SEDs of node versatility showed sharp sign variations across densities. This pronounced instability, likely attributable to the stochastic nature of the underlying Louvain algorithm used for module detection (Lancichinetti & Fortunato, 2009), indicates that $f_{mv}$ may be a less reliable metric for cognitive analysis in MIND. In contrast, $f_{mv}$ was negatively correlated with IQ in MSN and demonstrated greater stability across connection densities, suggesting a preliminary association where higher cognitive ability is associated with more specialized and less versatile structural connectivity. This finding offers initial support for the efficient modularity hypothesis (Bullmore & Sporns, 2012; Bassett et al., 2009; Puxeddu et al., 2020; Sporns, 2013), which posits that cognitive performance benefits from a more modular network organization, where information processing depends on efficient within-module integration rather than extensive cross-module communication. Such an organization is thought to enhance processing efficiency by reducing interference and maintaining functional specialization. This finding, however, contrasts with Solé-Casals et al. (2019), who reported higher mean versatility in GG using structural covariance networks. Methodological differences in network construction may account for this discrepancy.

- **The relationship between hemispheric connection and cognitive performance.**

Our analysis reveals distinct and network-specific patterns in the relationship between hemispheric connectivity and cognitive performance for MSN and MIND networks. While both are structural networks constructed with the same types of morphometric features, they capture different aspects of brain organization that manifest in divergent associations with cognitive abilities. In our data, hemispheric organization in MSNs shows consistent and interpretable patterns associated with group differences and individual cognitive abilities, whereas MIND networks exhibit more variable and less stable relationships.

- A fundamental difference emerges in baseline hemispheric organization: MSN networks show stronger right intra-hemispheric connections compared to left intra-hemispheric connections in both groups, aligning with findings from Jiang et al. (2019). In contrast, MIND networks exhibit the opposite pattern, with stronger left intra-hemispheric connections across both groups.
- In MSNs, we observed a potentially meaningful pattern of hemispheric specialization associated with higher cognitive performance. This pattern aligns with established theories of hemispheric specialization, which suggest that increased hemispheric independence throughout primate evolution contributed to advanced cognitive abilities (Rilling & Insel, 1999). Specifically, the consistently positive correlations between IQ and both left intra-hemispheric strength ($S_{LL}$) and the ratio of intra- to inter-hemispheric connections ($R_{ii}$) across different connection densities indicate a preliminary association where enhanced cognitive ability is associated with strengthened within-hemisphere connectivity, particularly in the left hemisphere. This interpretation is further supported by both larger Euclidean distances between groups and stronger positive correlations with IQ for left hemispheric measures. The negative correlation between IQ and inter-hemispheric strength ($S_{LR}$) additionally suggests that optimal cognitive performance might involve not only enhanced intra-hemispheric connectivity but also more selective cross-hemisphere communication. The group-level analysis provides further support for this interpretation, demonstrating that individuals with higher cognitive abilities exhibit stronger left intra-hemispheric connections alongside weaker right intra-hemispheric and inter-hemispheric connections compared to controls.
- In contrast, MIND networks show minimal group differences in hemispheric connection strengths despite high Euclidean distances in intra-hemispheric connections. The generally weak and unstable correlations between hemispheric metrics and IQ suggest that MIND networks may exhibit greater measurement instability and reduced effectiveness in capturing cognition-relevant hemispheric organization. This pronounced instability in MIND’s association with cognitive performance likely stems from methodological differences in network construction, particularly the use of distributional similarity measures compared to MSN’s correlation-based approach.

- **Limitations.**

While this study provides valuable insights into MSNs and MIND networks, several limitations should be acknowledged.

- First, the sample size of 29 male children is relatively small, limiting the generalizability of the findings to broader populations. It’s also worth noting the lack of diversity in terms of gender, age, and Catalan cultural context, which could restrict the identification of potential variations related to these factors in other cultural or educational settings. Future studies should aim to expand the cohorts to validate and deepen these preliminary findings.
- Second, the analyses were conducted using a single imaging modality (sMRI). Incorporating multimodal data, such as functional MRI or diffusion tensor imaging, could provide a more comprehensive understanding of brain network dynamics.
- Third, this study did not consider negative values in MSNs during connection density-related analyses and did not apply additional preprocessing steps to the brain networks. Different preprocessing methods, such as taking absolute values of MSNs, could alter the topological properties of networks and affect the results and their interpretations.

Addressing these limitations will improve the robustness and applicability of brain network research in understanding cognitive performance.

## Conclusion

This study systematically investigated the influence of connection density, topological features, and hemispheric connectivity on cognitive performance through comparative analysis of MSN and MIND networks. Several key conclusions can be drawn:

### Connection Density

We identified $p=0.05$ to 0.15 as the optimal density range for analyzing both MSN and MIND networks of 308 nodes. Within this range, the networks preserve biologically meaningful connections while demonstrating maximum group discrimination and stable and relatively strong correlation patterns with cognitive performance. However, MIND networks exhibited greater variability and less consistent correlation patterns across connection densities compared to MSNs.

### Topological Features

Advanced network segregation metrics (including transitivity, clustering coefficient, and local efficiency) and integration metrics (including participation coefficient and node versatility), along with their global summaries (e.g., mean clustering coefficient, global efficiency, mean participation coefficient, and mean versatility), proved more sensitive to cognitive differences for both networks. Higher cognitive ability was associated with more modular network organization and less versatile structural connectivity, particularly in MSNs. These metrics showed network-specific association patterns with cognitive performance, and the mean participation coefficient exhibited notable instability.

### Hemispheric Connectivity

MSNs revealed a robust pattern of hemispheric specialization linked to cognitive ability, characterized by stronger left intra-hemispheric connectivity and selective reduction in inter-hemispheric communication in higher performers. This aligns with established theories of hemispheric independence. In contrast, MIND networks failed to consistently capture hemispheric-cognition relationships, reflecting underlying methodological differences in network construction.

In summary, MSNs provide a more robust and reliable framework for analyzing structural brain-cognition relationships, demonstrating consistent patterns across connection density in topological and hemispheric dimensions, supporting the efficient modularity hypothesis and the hemispheric specialization theories. The insights enhance our understanding of brain-cognition relationships and provide practical guidelines for parameter selection, including optimal connection density thresholds and relevant metric identification in structural network analysis. Future studies should expand into more diverse populations and incorporate multimodal neuroimaging to further validate and generalize these results.

## Data Availability

No datasets were generated or analysed during the current study. The raw (anonymized) MRI data and the cortical thickness data are available in the OpenNeuro repository (https://openneuro.org/datasets/ds001988).

## References

[CR1] Alexander-Bloch, A. F., Gogtay, N., Meunier, D., Birn, R., Clasen, L., Lalonde, F., Lenroot, R., Giedd, J., & Bullmore, E. T. (2010). Disrupted modularity and local connectivity of brain functional networks in childhood-onset schizophrenia. *Frontiers in systems neuroscience,**4*, 147. 21031030 10.3389/fnsys.2010.00147 PMC2965020

[CR2] Bassett, D. S., Bullmore, E. T., Meyer-Lindenberg, A., Apud, J. A., Weinberger, D. R., & Coppola, R. (2009). Cognitive fitness of cost-efficient brain functional networks. *Proceedings of the National Academy of Sciences,**106*(28), 11747–11752. 10.1073/pnas.0903641106 PMC2703669 19564605

[CR3] Benjamini, Y., & Hochberg, Y. (1995). Controlling the false discovery rate: a practical and powerful approach to multiple testing. *Journal of the Royal statistical society: series B (Methodological),**57*(1), 289–300.

[CR4] Blondel, V. D., Guillaume, J.-L., Lambiotte, R., & Lefebvre., E. (2008). Fast unfolding of communities in large networks. *Journal of statistical mechanics: theory and experiment,**2008*(10), P10008.

[CR5] Brandes, U. (2001). A faster algorithm for betweenness centrality. *Journal of mathematical sociology,**25*(2), 163–177.

[CR6] Bullmore, E., & Sporns, O. (2012). The economy of brain network organization. *Nature reviews neuroscience,**13*(5), 336–349. 22498897 10.1038/nrn3214

[CR7] Chung, M. K. (2019). *Brain network analysis*. Cambridge University Press.

[CR8] Desikan, R. S., Ségonne, F., Fischl, B., Quinn, B. T., Dickerson, B. C., Blacker, D., Buckner, R. L., Dale, A. M., Maguire, R. P., Hyman, B. T. et al. An automated labeling system for subdividing the human cerebral cortex on mri scans into gyral based regions of interest. *Neuroimage*, *31*(3), 968–980. 10.1016/j.neuroimage.2006.01.021 16530430

[CR9] Fischl, B., & Dale, A. M. (2000). Measuring the thickness of the human cerebral cortex from magnetic resonance images. *Proceedings of the National Academy of Sciences,**97*(20), 11050–11055. 10.1073/pnas.200033797 PMC27146 10984517

[CR10] Fornito, A., Zalesky, A., & Bullmore, E. (2016). *Fundamentals of brain network analysis*. Academic press.

[CR11] Genon, S., Eickhoff, S. B., & Kharabian, S. (2022). Linking interindividual variability in brain structure to behaviour. *Nature Reviews Neuroscience,**23*(5), 307–318. 35365814 10.1038/s41583-022-00584-7

[CR12] Ghosh, S. S., Kakunoori, S., Augustinack, J., Nieto-Castanon, A., Kovelman, I., Gaab, N., Christodoulou, J. A., Triantafyllou, C., Gabrieli, J. D., & Fischl, B. (2010). Evaluating the validity of volume-based and surface-based brain image registration for developmental cognitive neuroscience studies in children 4 to 11 years of age. *Neuroimage,**53*(1), 85–93. 10.1016/j.neuroimage.2010.05.075 PMC2914629 20621657

[CR13] Guimera, R., & Nunes Amaral, L. A. (2005). Functional cartography of complex metabolic networks. *nature*, *433*(7028), 895–900. 10.1038/nature03288 PMC2175124 15729348

[CR14] Han, S., Sun, Z., Zhao, K., Duan, F., Caiafa, C. F., Zhang, Y., & Solé-Casals, J. (2024). Early prediction of dementia using fmri data with a graph convolutional network approach. *Journal of Neural Engineering,**21*(1), Article 016013. 10.1088/1741-2552/ad1e22 38215493

[CR15] Heinsfeld, A., Franco, A. R., Craddock, R. C., Buchweitz, A., & Meneguzzi, F. (2018). Identification of autism spectrum disorder using deep learning and the ABIDE dataset. *NeuroImage: Clinical*, 17, 16–23. 10.1016/j.nicl.2017.08.017 PMC5635344 29034163

[CR16] Hosseini, S. M. H., Hoeft, F., & Kesler, S. R. (2012). Gat: a graph-theoretical analysis toolbox for analyzing between-group differences in large-scale structural and functional brain networks. *PloS one,**7*(7), Article e40709. 10.1371/journal.pone.0040709 PMC3396592 22808240

[CR17] Janssen, J., Gallego, A. G., Martínez Díaz-Caneja, C., Gonzalez Lois, N., Janssen, N., González-Peñas, J., Macias Gordaliza, P., Buimer, E., van Haren, N., Arango, C., et al. (2025). Heterogeneity of morphometric similarity networks in health and schizophrenia. *Schizophrenia,**11*(1), 70. 40274815 10.1038/s41537-025-00612-2 PMC12022303

[CR18] Jeffreys, H. (1973). *Scientific inference*. Cambridge Universi.

[CR19] Jiang, X., Shen, Y., Yao, J., Zhang, L., Xu, L., Feng, R., Cai, L., Liu, J., Chen, W., & Wang, J. (2019). Connectome analysis of functional and structural hemispheric brain networks in major depressive disorder. *Translational psychiatry,**9*(1), 136. 30979866 10.1038/s41398-019-0467-9 PMC6461612

[CR20] Kong, X.-Z., Liu, Z., Huang, L., Wang, X., Yang, Z., Zhou, G., Zhen, Z., & Liu, J. (2015). Mapping individual brain networks using statistical similarity in regional morphology from mri. *PloS one,**10*(11), Article e0141840. 10.1371/journal.pone.0141840 PMC4633111 26536598

[CR21] Lancichinetti, A., & Fortunato, S. (2009). Community detection algorithms: a comparative analysis. *Physical Review E-Statistical, Nonlinear, and Soft Matter Physics,**80*(5), Article 056117. 10.1103/PhysRevE.80.056117 20365053

[CR22] Latora, V., & Marchiori, M. (2001). Efficient behavior of small-world networks. *Physical review letters,**87*(19), Article 198701. 10.1103/PhysRevLett.87.198701 11690461

[CR23] Li, J., Seidlitz, J., Suckling, J., Fan, F., Ji, G.-J., Meng, Y., Yang, S., Wang, K., Qiu, J., Chen, H., et al. (2021). Cortical structural differences in major depressive disorder correlate with cell type-specific transcriptional signatures. *Nature communications,**12*(1), 1647. 33712584 10.1038/s41467-021-21943-5 PMC7955076

[CR24] Liu, J., Li, M., Pan, Y., Lan, W., Zheng, R., Wu, F.-X., & Wang, J. (2017). Complex brain network analysis and its applications to brain disorders: a survey. *Complexity,**2017*(1), 8362741.

[CR25] Li, W., Yang, C., Shi, F., Wu, S., Wang, Q., Nie, Y., & Zhang, X. (2017). Construction of individual morphological brain networks with multiple morphometric features. *Frontiers in Neuroanatomy,**11*, 34. 28487638 10.3389/fnana.2017.00034 PMC5403938

[CR26] Lo, C.-Y.Z., He, Y., & Lin, C.-P. (2021). Graph theoretical analysis of human brain structural networks. *Reviews in the Neurosciences,**22*(5), 551–563. 10.1515/RNS.2011.039 21861783

[CR27] Newman, M. (2010). *Networks: An Introduction*. Oxford University Press.

[CR28] Newman, M. E. J. (2002). Assortative mixing in networks. *Physical review letters,**89*(20), Article 208701. 10.1103/PhysRevLett.89.208701 12443515

[CR29] Onnela, J.-P., Saramäki, J., Kertész, J., & Kaski., K. (2005). Intensity and coherence of motifs in weighted complex networks. *Physical Review E-Statistical, Nonlinear, and Soft Matter Physics,**71*(6), Article 065103. 10.1103/PhysRevE.71.065103 16089800

[CR30] Park, H.-J., & Friston, K. (2013). Structural and functional brain networks: from connections to cognition. *Science,**342*(6158), 1238411. 24179229 10.1126/science.1238411

[CR31] Pérez-Cruz, F. (2008). Kullback-leibler divergence estimation of continuous distributions. In: *2008 IEEE international symposium on information theory*, pp. 1666–1670. IEEE.

[CR32] Puxeddu, M. G., Faskowitz, J., Betzel, R. F., Petti, M., Astolfi, L., & Sporns, O. (2020). The modular organization of brain cortical connectivity across the human lifespan. *NeuroImage,**218*, Article 116974. 10.1016/j.neuroimage.2020.116974 32450249

[CR33] Qi, S., Meesters, S., Nicolay, K., ter Haar Romeny, B. M., & Ossenblok, P. (2015). The influence of construction methodology on structural brain network measures: A review. *Journal of neuroscience methods,**253*, 170–182. 26129743 10.1016/j.jneumeth.2015.06.016

[CR34] Rilling, J. K., & Insel, T. R. (1999). Differential expansion of neural projection systems in primate brain evolution. *Neuroreport,**10*(7), 1453–1459. 10380962 10.1097/00001756-199905140-00012

[CR35] Romero-Garcia, R., Atienza, M., Clemmensen, L. H., & Cantero, J. L. (2012). Effects of network resolution on topological properties of human neocortex. *Neuroimage,**59*(4), 3522–3532. 22094643 10.1016/j.neuroimage.2011.10.086

[CR36] Rubinov, M., & Sporns, O. (2010). Complex network measures of brain connectivity: uses and interpretations. *Neuroimage,**52*(3), 1059–1069. 19819337 10.1016/j.neuroimage.2009.10.003

[CR37] Sebenius, I., Dorfschmidt, L., Seidlitz, J., Alexander-Bloch, A., Morgan, S. E., & Bullmore., E. (2025). Structural mri of brain similarity networks. *Nature Reviews Neuroscience*, 26(1):42–59, 2025. 10.1038/s41583-024-00882-2 PMC12936990 39609622

[CR38] Sebenius, I., Seidlitz, J., Warrier, V., Bethlehem, R. A. I., Alexander-Bloch, A., Mallard, T. T., Garcia, R. R., Bullmore, E. T., & Morgan, S. E. (2023). Robust estimation of cortical similarity networks from brain mri. *Nature Neuroscience,**26*(8), 1461–1471. 37460809 10.1038/s41593-023-01376-7 PMC10400419

[CR39] Seidlitz, J., Váša, F., Shinn, M., Romero-Garcia, R., Whitaker, K. J., Vértes, P. E., Wagstyl, K., Reardon, P. K., Clasen, L., Liu, S., et al. (2018). Morphometric similarity networks detect microscale cortical organization and predict inter-individual cognitive variation. *Neuron,**97*(1), 231–247. 29276055 10.1016/j.neuron.2017.11.039 PMC5763517

[CR40] Shinn, M., Romero-Garcia, R., Seidlitz, J., Váša, F., Vértes, P. E., & Bullmore, E. (2017). Versatility of nodal affiliation to communities. *Scientific Reports,**7*(1), 4273. 28655911 10.1038/s41598-017-03394-5 PMC5487331

[CR41] Solé-Casals, J., Serra-Grabulosa, J. M., Romero-Garcia, R., Vilaseca, G., Adan, A., Vilaró, N., Bargalló, N., & Bullmore, E. T. (2019). Structural brain network of gifted children has a more integrated and versatile topology. *Brain Structure and Function,**224*(7), 2373–2383. 31250156 10.1007/s00429-019-01914-9

[CR42] Sporns, O. (2013). Network attributes for segregation and integration in the human brain. *Current opinion in neurobiology,**23*(2), 162–171. 23294553 10.1016/j.conb.2012.11.015

[CR43] Sun, Y., Chen, P., Liu, Y., & Zhao, K. (2024a). Macroscale brain structural network coupling is related to ad progression. In: *2024 IEEE International Symposium on Biomedical Imaging (ISBI)*, pp. 1–4. IEEE.

[CR44] Sun, K., Chen, G., Liu, C., Chu, Z., Huang, L., Li, Z., Zhong, S., Ye, X., Zhang, Y., Jia, Y. et al. (2024b). A novel msn-ii feature extracted from t1-weighted mri for discriminating between bd patients and mdd patients. *Journal of Affective Disorders*. 10.1016/j.jad.2024.11.047 39557301

[CR45] Sun, Y., Lee, R., Chen, Y., Collinson, S., Thakor, N., Bezerianos, A., & Sim, K. (2015) Progressive gender differences of structural brain networks in healthy adults: a longitudinal, diffusion tensor imaging study.015 *PloS one*, *10*(3), e0118857. 10.1371/journal.pone.0118857 PMC4350987 25742013

[CR46] Tewarie, P., van Dellen, E., Hillebrand, A., & Stam, C. J. (2015). The minimum spanning tree: an unbiased method for brain network analysis. *Neuroimage,**104*, 177–188. 25451472 10.1016/j.neuroimage.2014.10.015

[CR47] Van Wijk, B. C., Stam, C. J., & Daffertshofer, A. (2010). Comparing brain networks of different size and connectivity density using graph theory. *PloS one,**5*(10), Article e13701. 10.1371/journal.pone.0013701 PMC2965659 21060892

[CR48] Vuksanović, V. (2022) Brain morphometric similarity and flexibility. *Cerebral Cortex Communications*, *3*(3), tgac024. 10.1093/texcom/tgac024 PMC9283106 35854840

[CR49] Watts, D. J., & Strogatz, S. H. (1998). *Collective dynamics of small-world networks. nature,**393*(6684), 440–442. 9623998 10.1038/30918

[CR50] Xia, M., Wang, J., & He, Y. (2013). Brainnet viewer: a network visualization tool for human brain connectomics. *PloS one,**8*(7), Article e68910. 10.1371/journal.pone.0068910 PMC3701683 23861951

[CR51] Yu, K., Wang, X., Li, Q., Zhang, X., Li, X., & Li, S. (2018). Individual morphological brain network construction based on multivariate euclidean distances between brain regions. *Frontiers in human neuroscience,**12*, 204. 29887798 10.3389/fnhum.2018.00204 PMC5981802

[CR52] Zhang, J., Feng, F., Han, T. Y., Duan, F., Sun, Z., Caiafa, C. F., & Solé-Casals, J. (2021). A hybrid method to select morphometric features using tensor completion and f-score rank for gifted children identification. *Science China Technological Sciences,**64*(9), 1863–1871.

